# Gamma power abnormalities in a *Fmr1*-targeted transgenic rat model of fragile X syndrome

**DOI:** 10.1038/s41598-020-75893-x

**Published:** 2020-11-02

**Authors:** Naoki Kozono, Ai Okamura, Sokichi Honda, Mitsuyuki Matsumoto, Takuma Mihara

**Affiliations:** 1 Drug Discovery Research, Astellas Pharma Inc, 21 Miyukigaoka, Tsukuba, Ibaraki 305-8585 Japan; 2 Neuroscience, La Jolla Laboratory, Astellas Research Institute of America LLC, San Diego, CA USA

**Keywords:** Sensory processing, Neurophysiology, Biomarkers, Biological models, Electrophysiology

## Abstract

Fragile X syndrome (FXS) is characteristically displayed intellectual disability, hyperactivity, anxiety, and abnormal sensory processing. Electroencephalography (EEG) abnormalities are also observed in subjects with FXS, with many researchers paying attention to these as biomarkers. Despite intensive preclinical research using *Fmr1* knock out (KO) mice, an effective treatment for FXS has yet to be developed. Here, we examined *Fmr1*-targeted transgenic rats (*Fmr1*-KO rats) as an alternative preclinical model of FXS. We characterized the EEG phenotypes of *Fmr1*-KO rats by measuring basal EEG power and auditory steady state response (ASSR) to click trains of stimuli at a frequency of 10–80 Hz. *Fmr1*-KO rats exhibited reduced basal alpha power and enhanced gamma power, and these rats showed enhanced locomotor activity in novel environment. While ASSR clearly peaked at around 40 Hz, both inter-trial coherence (ITC) and event-related spectral perturbation (ERSP) were significantly reduced at the gamma frequency band in *Fmr1*-KO rats. *Fmr1*-KO rats showed gamma power abnormalities and behavioral hyperactivity that were consistent with observations reported in mouse models and subjects with FXS. These results suggest that gamma power abnormalities are a translatable biomarker among species and demonstrate the utility of *Fmr1*-KO rats for investigating drugs for the treatment of FXS.

## Introduction

Fragile X syndrome (FXS) is a debilitating neurodevelopmental disorder caused by a CGG repeat expansion mutation in the *fragile X mental retardation 1* (*FMR1)* gene on the X chromosome^1^ that results in loss of the fragile X mental retardation protein (FMRP). FXS is estimated to affect 1 in 4000 men and 1 in 8000 women^2^, and patients are characterized by intellectual disability, hyperactivity, anxiety, seizures, autism-like symptoms, and abnormal sensory processing^3,4^. Auditory processing deficits are also a common feature in subjects with FXS^5–8^. Further, electroencephalography (EEG) abnormalities are observed in 74% of males with FXS, and hyperactivity, a major behavioral symptom of FXS, is observed in 50–66%^9^. EEG abnormalities in subjects with FXS are usually characterized by an enhancement in the amplitude of the N1 of the event-related potential (ERP) in response to auditory stimuli^5,10–12^ and basal gamma power^6,13,14^. Furthermore, recent evidence suggests that cortical oscillatory activity contributes to sensory hypersensitivity and social communication deficits in FXS, and that auditory steady state response (ASSR) at gamma frequencies is reduced in FXS^6,13^, the abnormalities that is widely used as a translational biomarker in neuropsychiatric disorders such as schizophrenia^15^ and developmental disorders^16^.

*Fmr1* knock out (KO) mice have been used as a preclinical model of FXS for more than 20 years^17^. Complete loss of FMRP causes neuronal morphological alterations such as changes to spine shape and density^18,19^, and behavioral abnormalities such as hyperactivity and hypersensitivity to sensory stimuli^20,21^. Note that EEG abnormalities are observed in preclinical mice models. *Fmr1* KO mice show blunted ASSR at the gamma frequency band, like that observed in subjects with FXS^22^. Thus, EEG measures appear to be a useful translational biomarker for drug development for FXS.

Despite intensive research into the pathophysiology of FMRP for more than a decade, there are yet to be any effective treatments of FXS patients^17^. Over 10 diverse interventions have failed or showed only minimal effects in clinical trials, despite preclinical studies having indicated various benefits of these interventions in *Fmr1* KO mice^23^. Thus, evidence from *Fmr1* KO mice alone may not be sufficient to warrant clinical investigation. Lessons learned from past drug developments for FXS suggest the need for further research into translatable biomarkers using mice of different genetic backgrounds (C57BL/6 or FVB) or other disease models^16,17,23^.

In 2014, *Fmr1*-targeted transgenic rats (*Fmr1*-KO rats) were established by Sage Laboratories, LLC (Emmett, ID, USA), using zinc finger nuclease (ZFN) technology to target exon 8 of the *Fmr1* gene^21^. ZFN pairs targeting the CATGAACAGTTTATCgtacgaGAAGATCTGATGGGT sequence in the *Fmr1* gene led to a 122 bp deletion that caused skipping of exon 8 and decreased Fmrp expression^24^. *Fmr1*-KO rats have been reported to display disrupted cortical processing of auditory stimuli^25^ and memory impairment based on hippocampal cellular and synaptic deficits^18^. For drug development in general, rat model can provide beneficial information to evaluate the safety risk of drug candidates compared with mouse model since toxicity studies are usually conducted with rats. However, reports on other characteristics of *Fmr1*-KO rats remain limited.

In present study, we examined EEG phenotypes, such as basal EEG power and ASSR, in *Fmr1*-KO rats and conducted a brief behavioral assessment to examine its utility as an alternative preclinical model for drug development in FXS. ASSR is an electrophysiological response entrained to both frequency and phase of rapid, periodic acoustic stimuli^26,27^. The entrainment is generally defined as two indices with time–frequency decomposition; inter-trial coherence (ITC) indicating phase consistency across trials and event-related spectral perturbation (ERSP) indicating event-related alteration of EEG frequency spectrum as a function of time. Accumulated evidence demonstrates the excellent test–retest reliability of ASSR in clinical setting, indicating the use of this method for drug development trial^28–31^. We employed the methodology of ASSR with ERSP and ITC that we recently established in rodent model^32^.

## Results

### *Fmr1*-KO rats display hyperactivity in a novel environment

**Figure 1:**
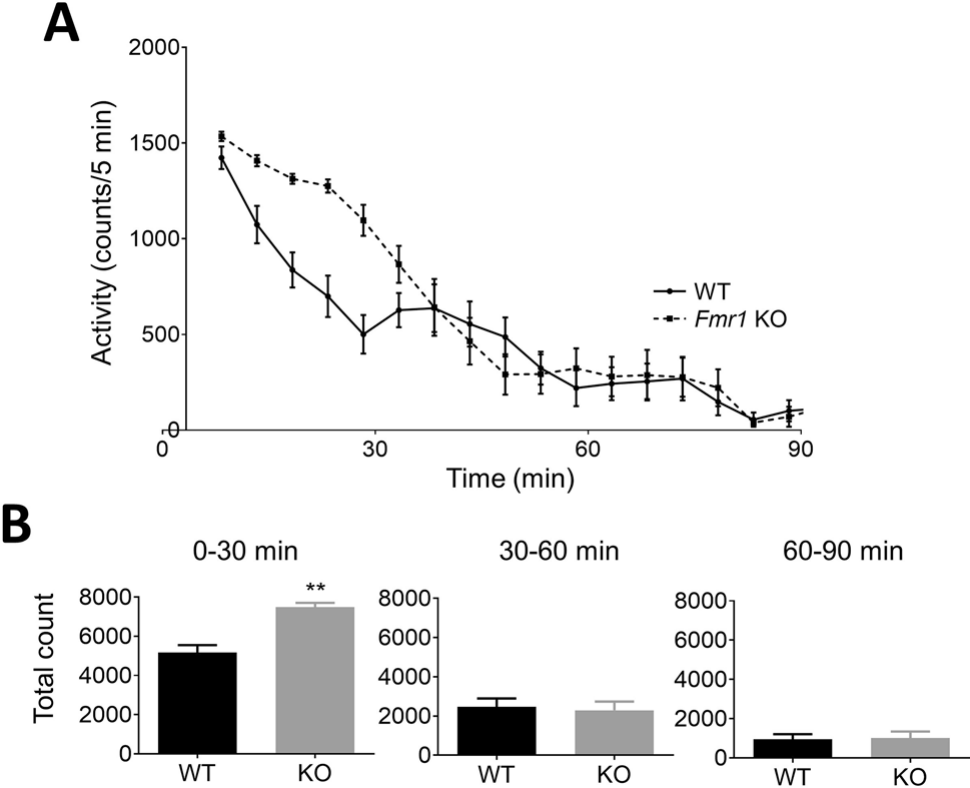
Locomotor activity in *Fmr1*-KO and WT rats in a novel environment. (**A**) Time course of locomotor activity (counts/5 min) in WT (solid line) and *Fmr1*-KO rats (dashed line) across 90 min after placement in a novel cage. (**B**) Total activity counts at 3 time periods (0–30, 30–60, and 60–90 min) in WT and *Fmr1*-KO rats. Data represent mean ± SEM (n = 13–14). **P < 0.01, significant differences between groups; unpaired *t*-test.

WT and *Fmr1*-KO rats displayed a time-dependent decrease in activity counts during the 90-min test period in novel cages (Fig. 1A). In the first 30 min, *Fmr1*-KO rats showed a significant increase in total activity counts compared with WT rats (Fig. 1B). In contrast, in the 30–60 min and 60–90 min time periods, total activity counts were not significantly different between *Fmr1*-KO rats and WT rats.

### *Fmr1*-KO rats display enhanced gamma power

**Figure 2:**
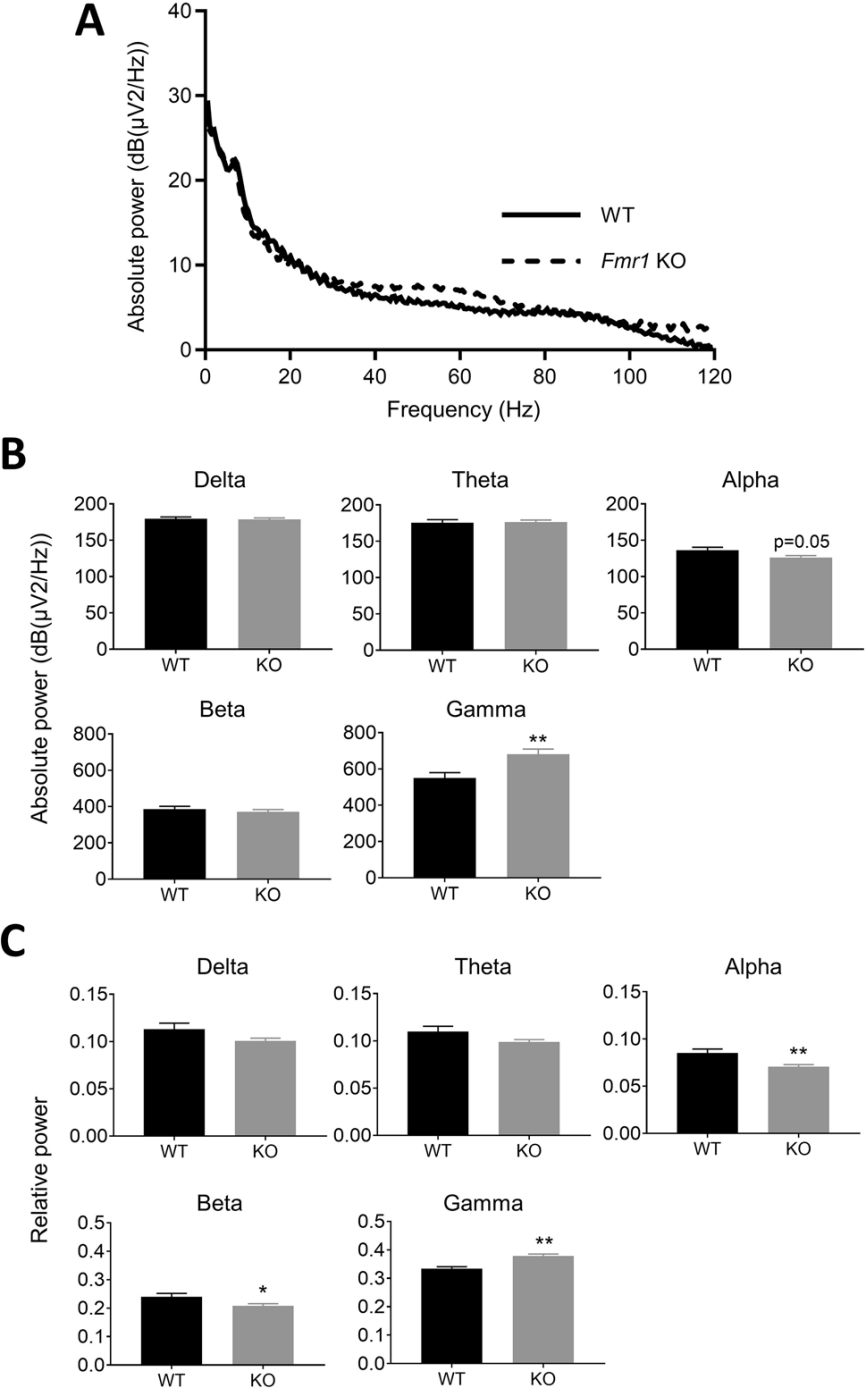
Comparison of basal power between *Fmr1*-KO and WT rats. (**A**) Spectra of baseline absolute power between 0 and 120 Hz in WT (solid line) and *Fmr1*-KO rats (dashed line). (**B**) Absolute power at delta (0.5–4 Hz), theta (4–8 Hz), alpha (8–12 Hz), beta (12–30 Hz), and gamma (30–80 Hz) frequency bands in WT and *Fmr1*-KO rats. (**C**) Relative power at delta, theta, alpha, beta, and gamma frequency bands in WT and *Fmr1*-KO rats. Data represent mean ± SEM (n = 13–14). *P < 0.05, **P < 0.01, significant differences between groups; unpaired *t*-test.

Power spectrum analysis was calculated from baseline power with no sound stimuli. Spectra of absolute power at around 40–80 Hz was higher in *Fmr1*-KO rats than in WT rats (Fig. 2A). Absolute power at the gamma frequency band (30–80 Hz) was significantly increased in *Fmr1*-KO rats compared with WT rats, while that at the alpha frequency band (8–12 Hz) tended to be decreased (p = 0.05) (Fig. 2B). Relative power at the gamma frequency band was significantly increased in *Fmr1*-KO rats compared with WT rats (Fig. 2C). In contrast, relative power at alpha and beta frequency bands (12–30 Hz) was significantly decreased in *Fmr1*-KO rats. Absolute and relative power at delta (0.5–4 Hz) and theta frequency bands (4–8 Hz) were not significantly different between *Fmr1*-KO and WT rats.

### Gamma synchronization of ITC and ERSP is reduced in *Fmr1*-KO rats

**Figure 3:**
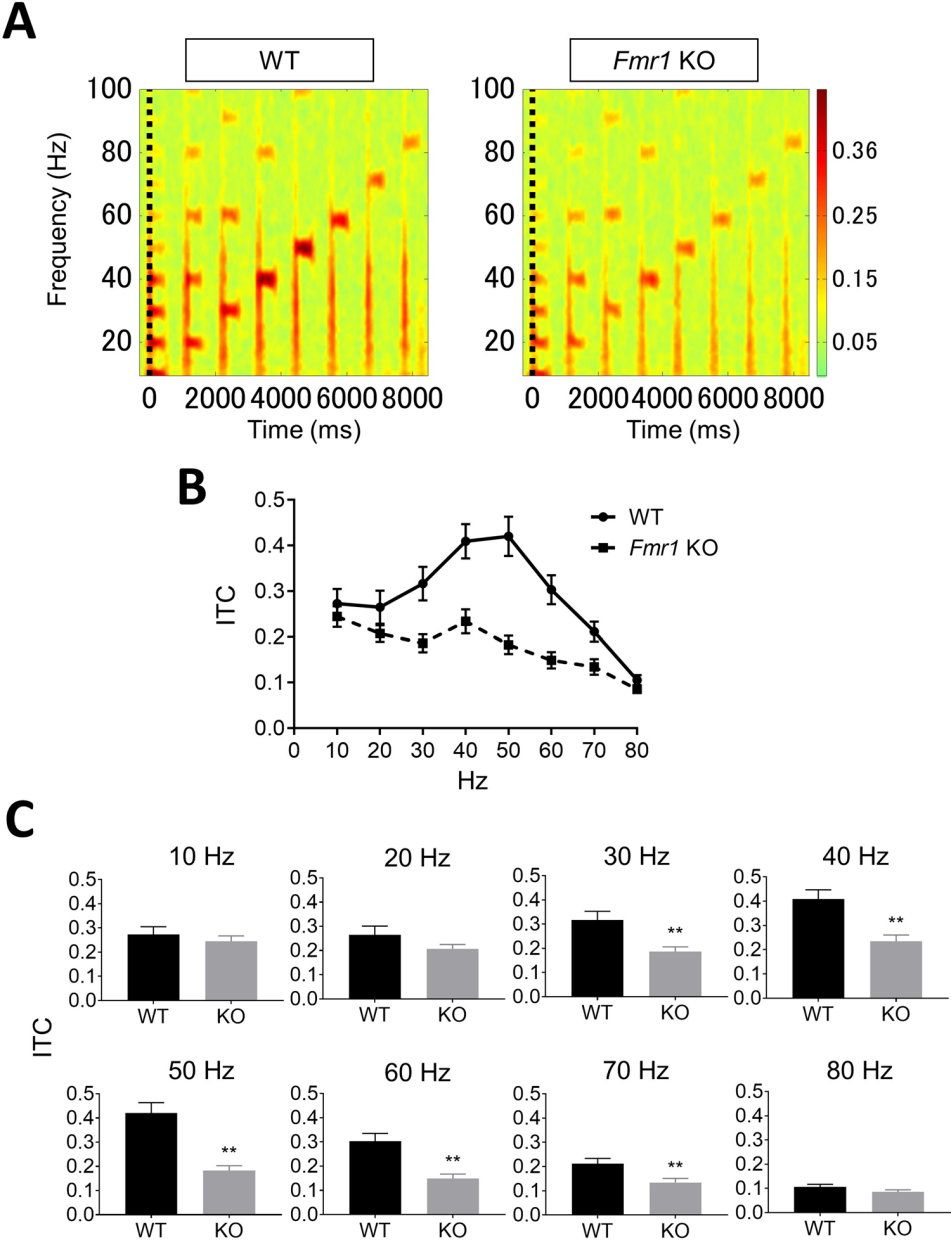
Comparison of ITC between *Fmr1*-KO and WT rats. (**A**) Time–frequency plots of ITC in WT and *Fmr1*-KO rats evoked by auditory click stimuli from 10 to 80 Hz. (**B**) Average of all ITC measured in WT (circle, solid line) and *Fmr1*-KO (square, dashed line) rats. (**C**) ITC in WT and *Fmr1*-KO rats at each frequency. Data represent mean ± SEM (n = 13–14). **P < 0.01, significant differences between groups; unpaired *t*-test.

**Figure 4:**
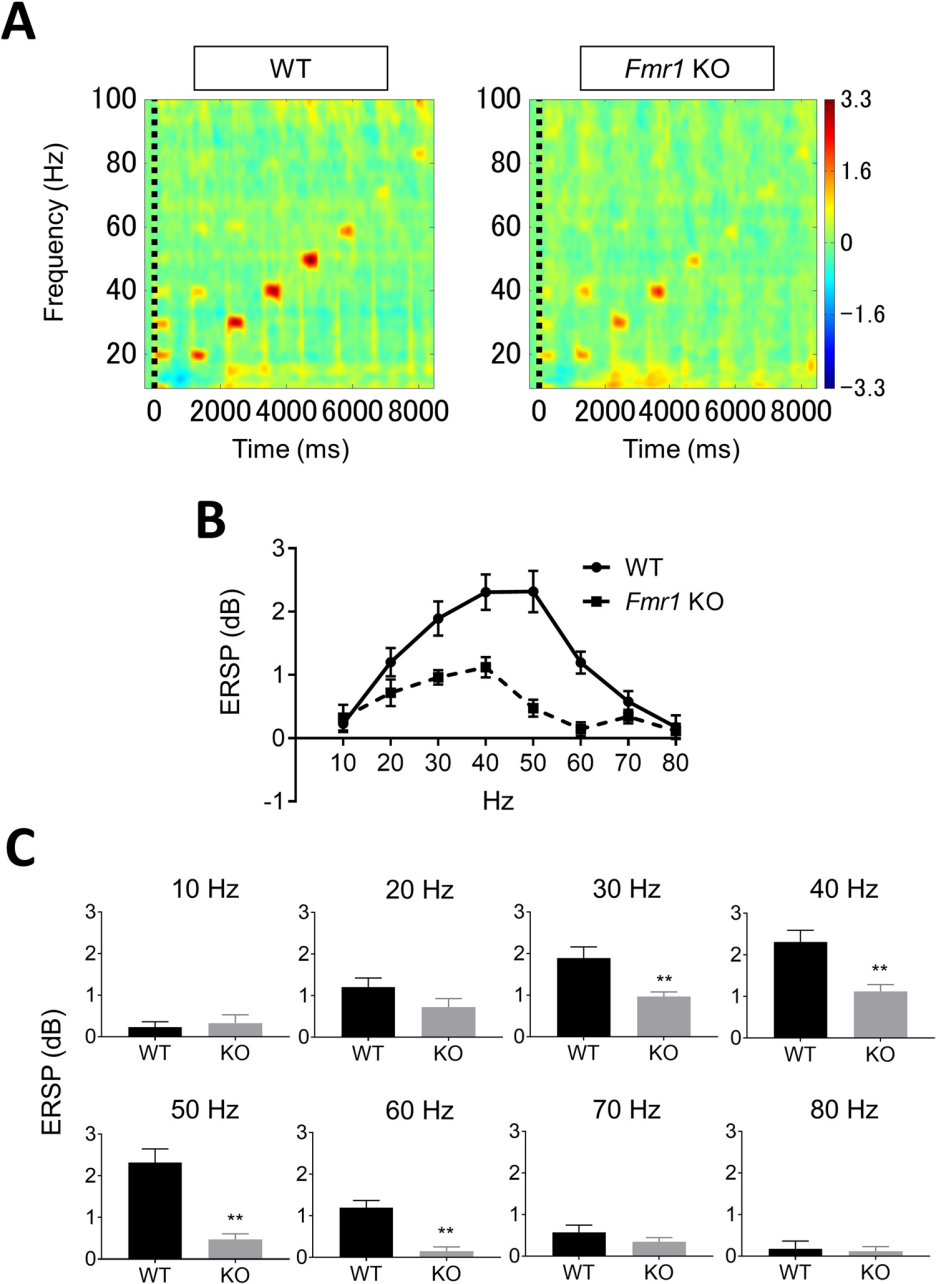
Comparison of ERSP between *Fmr1*-KO and WT rats. (**A**) Time–frequency plots of ERSP in WT and *Fmr1*-KO rats evoked by auditory click stimuli from 10 to 80 Hz. (**B**) Average of all ERSP measured in WT (circle, solid line) and *Fmr1*-KO (square, dashed line) rats. (**C**) ERSP in WT and *Fmr1*-KO rats at each frequency. Data represent mean ± SEM (n = 13–14). **P < 0.01, significant differences between groups; unpaired *t*-test.

ITC analysis showed that auditory click-train stimuli elicited frequency-dependent responses from 10 to 80 Hz in both *Fmr1*-KO and WT rats (Fig. 3A). In the gamma frequency band (30–80 Hz) for ASSR, ITC reached a maximum at around 40–50 Hz in both *Fmr1*-KO and WT rats (Fig. 3B). ITC was significantly decreased at 30, 40, 50, 60, and 70 Hz in *Fmr1*-KO rats compared with WT rats. Meanwhile, there were no significant differences in ITC at 10, 20, 80 Hz between genotypes (Fig. 3C). In ERSP analysis, auditory click-train stimuli similarly elicited frequency-dependent responses in both *Fmr1*-KO and WT rats (Fig. 4A). *Fmr1*-KO rats showed lower ERSP than WT rats, although a peak response was observed at 40–50 Hz within the gamma frequency band in both *Fmr1*-KO and WT rats (Fig. 4B). ERSP was significantly decreased at 30, 40, 50, and 60 Hz in *Fmr1*-KO rats compared with WT rats. In contrast, there was no significant difference in ERSP at 10, 20, 70 and 80 Hz between *Fmr1*-KO and WT rats (Fig. 4C).

## Discussion

While the behavioral and neurophysiological characterizations of *Fmr1* KO mice have been extensively replicated, those of *Fmr1*-KO rats remain limited. The present study is the first to describe the neurophysiological phenotypes of *Fmr1*-KO rats.

First, we assessed basal EEG profiles in *Fmr1*-KO rats. *Fmr1*-KO rats displayed significantly augmented baseline gamma power compared to WT but decreased alpha power. This pattern of baseline EEG power in *Fmr1*-KO rats is consistent with findings in subjects with FXS, who show augmented power in the gamma frequency band, but reduced power in the alpha frequency band^8,13^. This augmented gamma power is also consistent with studies in *Fmr1* KO mice^22,33^, which display augmented neuronal excitability related to alterations in input to fast spiking inhibitory interneurons synchronized to the gamma frequency band^34,35^. Alpha activity reportedly produces bouts of inhibition that repeat every 100 ms, and these alpha oscillations modulate gamma activity driven by GABAergic inhibitory activity in sensory information processing^36–38^. This pattern of increased gamma and decreased alpha power suggests the presence of impaired gamma activity with involvement from GABAergic inhibitory neurons in *Fmr1*-KO rats. In this study, we did not observe any alterations in power in the theta frequency band, although power in the beta frequency band was reduced in *Fmr1*-KO rats. Subjects with FXS show enhanced basal theta power and no change in power in the beta frequency band^8,13^. Resting power in the theta and beta frequency bands are not changed in *Fmr1* KO mice^22^. The discordance in theta and beta frequency bands between rodents and subjects with FXS is important to note and requires further study. Nevertheless, our finding that basal power is enhanced in the gamma frequency band in a preclinical rat model of FXS is consistent with the EEG phenotypes observed in *Fmr1* KO mice and subjects with FXS.

Enhanced neuronal excitability is associated with behavioral symptoms such as increased anxiety and locomotor activity^9,39,40^, some of the most consistent behavioral symptoms observed in subjects with FXS^41^. The present study found that *Fmr1*-KO rats showed enhanced locomotor activity for the first 30 min after placement in a novel environment but not at 30–60 min or 60–90 min. Studies using *Fmr1* KO mice have reported that the hyperactivity was a reaction to novelty^42,43^. Our results from *Fmr1*-KO rats suggest that the hyperactivity may be a reaction to novelty, because the significant augmentation in locomotor activity was only observed for the first 30 min after placement in a novel testing field. Interestingly, startle response to sounds of collision between pedestal and top cage was observed in *Fmr1*-KO rats during measurement of locomotor activity only in *Fmr1*-KO not wild type rats (data not shown). Sensory hypersensitivity is a common phenotype in subjects with FXS^44^, with habituation deficits to repeated sounds, especially N1 amplitude of ERP^5–7^. Further research of ERP responses and habituation to repeated sounds stimuli is needed. In vitro slice recordings have shown increased excitability in excitatory neurons from the somatosensory cortex of *Fmr1* KO mice^45^. Specific deletion of forebrain excitatory neurons in FMRP increases abnormal locomotor activity^46^. Taken together, these findings on basal power and hyperactivity suggest that *Fmr1*-KO rats exhibit cortical hyper-excitability as a pathophysiological consequence of FMRP deletion.

Second, we provide the first report of ASSR in *Fmr1*-KO rats. WT and *Fmr1*-KO rats showed clear ITC and ERSP responses at 10–80 Hz. Peak ITC and ERSP responses observed at 40–50 Hz in the gamma frequency band (30–80 Hz) are consistent with previous reports in rats^32^ and humans^15,47,48^. ASSR recordings revealed clear impairment of ITC at 30–70 Hz in *Fmr1*-KO rats compared with WT rats. Additionally, *Fmr1*-KO rats demonstrated increased basal power in the gamma frequency band. A similar impairment of the phase-locking factor of ASSR at only 30–58 Hz was observed in subjects with FXS, with phase-locking abnormalities reported to be associated with increased gamma single-trial power^14^. Further, there was also clear impairment of ERSP at 30–60 Hz in *Fmr1*-KO rats. We used a previously developed ASSR paradigm that adopted click sounds as stimuli in freely moving rats^32^, with a similar method having been used in subjects with schizophrenia^49^. However, no robust impairment of ERSP in the gamma frequency band has been observed in subjects with FXS or *Fmr1* KO mice. It is important to note that these studies used “chirp” stimuli to elicit ASSR^14,22^. Although the differences associated with using “click” and “chirp” stimuli for eliciting ASSR remain elusive, the click auditory stimulus has been reported to induce large effect sizes and deficits in ASSR evoked by 40-Hz click stimulation have been used as a translational biomarker for schizophrenia^15,50,51^. These findings may support the use of click-stimuli-evoked ASSR as a robust method for determining ITC and ERSP in *Fmr1*-KO rats. Taken together, the specific attenuation of ASSR at 30–60 Hz in *Fmr1*-KO rats as it is in *Fmr1* KO mice and subjects with FXS suggests that ASSR in the gamma frequency band is a biomarker for FXS.

*Fmr1*-KO rats exhibited ASSR attenuation and enhanced basal power specific to gamma frequency band, these similar EEG features were observed in *Fmr1* KO mice and subjects with FXS^13,14,22^. Preclinical research in *Fmr1* KO mice has shown that increased gamma excitability decreases excitatory drive in fast-spiking inhibitory interneurons, resulting in increased and poorly synchronized pyramidal cell firing in the gamma frequency range^52^. *Fmr1*-KO rats show deficits of switching process from an elevated gamma state to a reduced gamma state with insufficiently synchronization related to firing rate of fast-spiking interneurons in visual cortex, and disrupted cortical processing of auditory stimuli^25,53,54^. These findings suggest that gamma power abnormalities with gamma band-ASSR attenuation and augmented baseline gamma power could attribute to imbalance between excitation and inhibition of the neural network in underlying mechanism of FXS.

## Methods

### Animals

Five-week-old *Fmr1*-KO and wild type (WT) rats were purchased from Oriental Yeast Co., Ltd. (Tokyo, Japan). *Fmr1*-KO rats were generated using the ZFN method^21^, and the lines were first generated on an outbred Sprague–Dawley background at Sage Laboratories, LLC.

### Surgery

Surgical operations were conducted in 13 WT and 14 *Fmr1*-KO male rats at 7–8 weeks of age to implant 4 electrodes under anesthesia with 2–2.5% isoflurane. A recording electrode was embedded onto the surface of the cortex using a customized switchable pedestal and electrode cables (S.E.R. Corporation, Tokyo, Japan). This paradigm using a switchable pedestal was previously developed to enable ECoG recordings to be taken from the parietal and temporal cortex in freely moving rats^32^. Briefly, the recording electrodes were placed at AP − 1.0 mm/ML − 1.0 mm (parietal cortex) and AP − 4.5 mm/ML − 7.5 mm/VD − 4.0 mm (temporal cortex) relative to bregma. Reference and ground electrodes were placed at AP 8.0 mm/ML − 1.5 mm and AP − 10.0 mm/ML − 1.5 mm, respectively. The switchable pedestal with stainless steel wire was attached to the cranium using methacrylic resin (GC Corporation, Tokyo, Japan). Rats were allowed to recover for approximately 14 days before testing. The rats were housed in groups until electrode implantation and then alone after implantation in ventilated cages under a 12-h light/dark cycle with food and water available ad libitum. All animal experimental procedures were performed in accordance with the Guide for the Care and Use of Laboratory Animals, 8th edition and approved by the Institutional Animal Care and Use Committee of Astellas Pharma Inc. Furthermore, Astellas Pharma Inc., Tsukuba Research Center was awarded Accreditation Status by the AAALAC International.

### Spontaneous locomotor activity

Locomotor activity was evaluated in rats implanted with a switchable pedestal at 9 weeks old. First, the rats were acclimated to the test room under 1 lx of red right. After 1 h of acclimation, test animals were moved from their home cages to new cages exposed to over 300 lx of light. Motor activity was measured for 90 min using a Supermex sensor (Muromachi inc., Tokyo, Japan) comprising paired infrared pyroelectric detectors that measure radiated body heat, and analyzed using CompACT AMS, ver. 3.82 (Muromachi Inc.). These data were divided into 3 time periods (0–30, 30–60, 60–90 min) and the total count was calculated for each time period.

### EEG recording

EEG experiments were performed according to previously reported methods^32^. Electrophysiological activity was recorded inside a Faraday cage with speakers attached to the top of the cage, using a high-impedance differential AC amplifier (model #1800, A-M Systems, Carlsbrog, WA, USA) and Spike 2 (Cambridge Electronic Design; CED, Cambridge, UK) with CED1401 (CED). All EEG experiments were performed in dim light (< 300 lx). All animals were implanted with electrodes connected to an amplifier via electrode cables during habituation and recording, and were allowed to move freely. Data from Spike2 were converted using EEGLAB in the MATLAB toolbox (Math Works, Natick MA, USA). The EEG measurements were conducted at 10 weeks of age in all animals.

Measurement of power spectrum was conducted under a series of 200-paired white-noise stimuli presented 500 ms apart at 85 sound decibels (dB) (10 ms in duration) with 9- to 13-s randomized interstimulus intervals. Acoustic stimuli were presented using Spike 2 with CED 1401 for another experimental purpose. Single-trial epochs between − 1 and 2 s relative to the first click were extracted from continuous data. Analysis and calculations were performed using Fourier transformation (FFT) analysis in the MATLAB toolbox. Absolute power was calculated at delta (0.5–4 Hz), theta (4–8 Hz), alpha (8–12 Hz), beta (12–30 Hz) and gamma (30–80 Hz) frequency bands. Relative values were calculated as the proportion of absolute power at each frequency band relative to total power (0–120 Hz).

For ASSR recording, auditory stimuli consisted of click sounds (80 sound dB) that included 500-ms trains at 10, 20, 30, 40, 50, 60, 70, and 80 Hz within a single train, as reported in our previous study^32^. These stimuli were repeated 200 times/trial with an inter-trial interval (ITI) and inter-signal interval (ISI) of 600 ms. Each sound stimulus was generated using Spike2 with CED 1401. For analysis of ASSR, a low-pass filter (100 Hz) was applied to the EEG data to remove artifacts. The time–frequency decomposition during each click sound trains were calculated by wavelet transformation (frequency limit: 8–100 Hz, wavelet cycle: 10–62.5, epoch size: 9.0 s). As output data, measurable factors were divided into two groups: event-related spectral perturbation (ERSP) (baseline: pre-stimulations from − 600 to 0 ms) and inter-trial coherence (ITC) in the MATLAB toolbox.

### Statistics

Unpaired t-test was used to detect significant differences between *Fmr1*-KO and WT rats. Statistical analyses were conducted using GraphPad Prism, version 8.0.2 for Windows (GraphPad Software, San Diego CA, USA, https://www.graphpad.com). P values < 0.05 were considered statistically significant.

## Conclusions

The present study is the first report to investigate ASSR in a rat model of FXS along with locomotor activity and basal EEG power. *Fmr1*-KO rats displayed reduced basal EEG power at the alpha frequency band, enhanced power at the gamma frequency band, and hyperactivity, consistent with findings in subjects with FXS and *Fmr1* KO mice. Moreover, ASSR at 10–80 Hz peaked at 40 Hz and robust attenuation of ITC and ERSP was observed at 30–60 Hz in *Fmr1*-KO rats. ASSR at the gamma frequency band, along with abnormal basal EEG properties and hyperactivity, may be a translational biomarker across species. Taken together, our findings indicate that *Fmr1*-KO rats are a useful preclinical model of FXS, and will aid in the acceleration of drug development by testing drug candidates with synergistic gamma power analysis among various species^28,33,55^.

## References

[1] Verkerk AJMH (1991). Identification of a gene (FMR-1) containing a CGG repeat coincident with a breakpoint cluster region exhibiting length variation in fragile X syndrome. Cell.

[2] Turner G, Webb T, Wake S, Robinson H (1996). Prevalence of fragile X syndrome. Am. J. Med. Genet..

[3] Lee AW, Ventola P, Budimirovic D, Berry-Kravis E, Visootsak J (2018). Clinical development of targeted fragile x syndrome treatments: an industry perspective. Brain Sci..

[4] Hagerman RJ (2017). Fragile X syndrome. Nat. Rev. Dis. Primers.

[5] Castrén M, Pääkkönen A, Tarkka IM, Ryynänen M, Partanen J (2003). Augmentation of auditory N1 in children with fragile x syndrome. Brain Topogr..

[6] Ethridge LE (2016). Reduced habituation of auditory evoked potentials indicate cortical hyper-excitability in Fragile X Syndrome. Transl. Psychiatry.

[7] Schneider A (2013). Electrocortical changes associated with minocycline treatment in fragile X syndrome. J. Psychopharmacol.

[8] Van der Molen MJ, Van der Molen MW (2013). Reduced alpha and exaggerated theta power during the resting-state EEG in fragile X syndrome. Biol. Psychol..

[9] Ciaccio C (2017). Fragile X syndrome: a review of clinical and molecular diagnoses. Ital. J. Pediatr..

[10] Knoth IS, Vannasing P, Major P, Michaud JL, Lippe S (2014). Alterations of visual and auditory evoked potentials in fragile X syndrome. Int. J. Dev. Neurosci..

[11] Van der Molen MJ (2012). Auditory and visual cortical activity during selective attention in fragile X syndrome: a cascade of processing deficiencies. Clin. Neurophysiol.

[12] Van der Molen MJ (2012). Auditory change detection in fragile X syndrome males: a brain potential study. Clin. Neurophysiol..

[13] Wang J (2017). A resting EEG study of neocortical hyperexcitability and altered functional connectivity in fragile X syndrome. J. Neurodev. Disord..

[14] Ethridge LE (2017). Neural synchronization deficits linked to cortical hyper-excitability and auditory hypersensitivity in fragile X syndrome. Mol. Autism.

[15] O’Donnell BF (2013). The auditory steady-state response (ASSR): a translational biomarker for schizophrenia. Suppl. Clin. Neurophysiol..

[16] Ethridge LE (2019). Auditory EEG biomarkers in fragile X syndrome: clinical relevance. Front. Integr. Neurosci..

[17] Dahlhaus R (2018). Of men and mice: modeling the fragile x syndrome. Front. Mol. Neurosci..

[18] Till SM (2015). Conserved hippocampal cellular pathophysiology but distinct behavioural deficits in a new rat model of FXS. Hum. Mol. Genet..

[19] Grossman AW, Elisseou NM, McKinney BC, Greenough WT (2006). Hippocampal pyramidal cells in adult Fmr1 knockout mice exhibit an immature-appearing profile of dendritic spines. Brain Res..

[20] Kazdoba TM, Leach PT, Silverman JL, Crawley JN (2014). Modeling fragile X syndrome in the Fmr1 knockout mouse. Intract. Rare Dis. Res..

[21] Hamilton SM (2014). Fmr1 and Nlgn3 knockout rats: novel tools for investigating autism spectrum disorders. Behav. Neurosci..

[22] Lovelace JW, Ethell IM, Binder DK, Razak KA (2018). Translation-relevant EEG phenotypes in a mouse model of Fragile X Syndrome. Neurobiol. Dis..

[23] Erickson CA (2017). Fragile X targeted pharmacotherapy: lessons learned and future directions. J. Neurodev. Disord..

[24] Golden CEM (2019). Deletion of the KH1 domain of Fmr1 leads to transcriptional alterations and attentional deficits in rats. Cereb. Cortex.

[25] Engineer CT (2014). Degraded speech sound processing in a rat model of fragile X syndrome. Brain Res.

[26] Galambos R, Makeig S, Talmachoff PJ (1981). A 40-Hz auditory potential recorded from the human scalp. Proc. Natl. Acad. Sci. U.S.A..

[27] Brenner CA (2009). Steady state responses: electrophysiological assessment of sensory function in schizophrenia. Schizophr. Bull..

[28] Honda S, Matsumoto M, Tajinda K, Mihara T (2020). Enhancing clinical trials through synergistic gamma power analysis. Front. Psychiatry.

[29] Roach BJ, D'Souza DC, Ford JM, Mathalon DH (2019). Test-retest reliability of time-frequency measures of auditory steady-state responses in patients with schizophrenia and healthy controls. Neuroimage Clin..

[30] Legget KT, Hild AK, Steinmetz SE, Simon ST, Rojas DC (2017). MEG and EEG demonstrate similar test-retest reliability of the 40 Hz auditory steady-state response. Int. J. Psychophysiol..

[31] McFadden KL (2014). Test-retest reliability of the 40 Hz EEG auditory steady-state response. PLoS ONE.

[32] Kozono N (2019). Auditory steady state response; nature and utility as a translational science tool. Sci. Rep..

[33] Sinclair, D. *et al.* GABA-B agonist baclofen normalizes auditory-evoked neural oscillations and behavioral deficits in the Fmr1 knockout mouse model of fragile x syndrome. *eNeuro* **4**, doi:10.1523/ENEURO.0380-16.2017 (2017). 10.1523/ENEURO.0380-16.2017 PMC5394929 28451631

[34] Cea-Del Rio CA, Huntsman MM (2014). The contribution of inhibitory interneurons to circuit dysfunction in Fragile X Syndrome. Front. Cell Neurosci..

[35] Salkoff DB, Zagha E, Yuzgec O, McCormick DA (2015). Synaptic mechanisms of tight spike synchrony at gamma frequency in cerebral cortex. J. Neurosci..

[36] Jensen O, Mazaheri A (2010). Shaping functional architecture by oscillatory alpha activity: gating by inhibition. Front. Hum. Neurosci..

[37] Mathewson KE (2011). Pulsed out of awareness: EEG alpha oscillations represent a pulsed-inhibition of ongoing cortical processing. Front. Psychol..

[38] Mazaheri A, Jensen O (2010). Rhythmic pulsing: linking ongoing brain activity with evoked responses. Front. Hum. Neurosci..

[39] Braat S, Kooy RF (2015). The GABAA receptor as a therapeutic target for neurodevelopmental disorders. Neuron.

[40] Penagarikano O, Mulle JG, Warren ST (2007). The pathophysiology of fragile x syndrome. Annu. Rev. Genomics Hum. Genet..

[41] Tranfaglia MR (2011). The psychiatric presentation of fragile x: evolution of the diagnosis and treatment of the psychiatric comorbidities of fragile X syndrome. Dev. Neurosci..

[42] Carreno-Munoz MI (2018). Potential involvement of impaired BKCa channel function in sensory defensiveness and some behavioral disturbances induced by unfamiliar environment in a mouse model of fragile x syndrome. Neuropsychopharmacology.

[43] Kramvis I, Mansvelder HD, Loos M, Meredith R (2013). Hyperactivity, perseveration and increased responding during attentional rule acquisition in the Fragile X mouse model. Front. Behav. Neurosci..

[44] Tsiouris JA, Brown WT (2004). Neuropsychiatric symptoms of fragile x syndrome. CNS Drugs.

[45] Hays SA, Huber KM, Gibson JR (2011). Altered neocortical rhythmic activity states in Fmr1 KO mice are due to enhanced mGluR5 signaling and involve changes in excitatory circuitry. J. Neurosci..

[46] Lovelace JW (2019). Deletion of Fmr1 from forebrain excitatory neurons triggers abnormal cellular, EEG, and behavioral phenotypes in the auditory cortex of a mouse model of fragile x syndrome. Cereb. Cortex.

[47] Puvvada KC (2018). Delta vs gamma auditory steady state synchrony in schizophrenia. Schizophr. Bull.

[48] Artieda J (2004). Potentials evoked by chirp-modulated tones: a new technique to evaluate oscillatory activity in the auditory pathway. Clin. Neurophysiol..

[49] Tada M (2016). Differential alterations of auditory gamma oscillatory responses between pre-onset high-risk individuals and first-episode schizophrenia. Cereb. Cortex.

[50] Griskova-Bulanova I (2018). 40Hz auditory steady-state response in schizophrenia: Sensitivity to stimulation type (clicks versus flutter amplitude-modulated tones). Neurosci. Lett..

[51] Thune H, Recasens M, Uhlhaas PJ (2016). The 40-Hz auditory steady-state response in patients with schizophrenia: a meta-analysis. JAMA Psychiatry.

[52] Gibson JR, Bartley AF, Hays SA, Huber KM (2008). Imbalance of neocortical excitation and inhibition and altered UP states reflect network hyperexcitability in the mouse model of fragile X syndrome. J. Neurophysiol..

[53] Berzhanskaya J (2017). Disrupted cortical state regulation in a rat model of Fragile X Syndrome. Cereb. Cortex.

[54] Berzhanskaya J, Phillips MA, Shen J, Colonnese MT (2016). Sensory hypo-excitability in a rat model of fetal development in Fragile X Syndrome. Sci. Rep.

[55] Yamazaki M, Honda S, Tamaki K, Irie M, Mihara T (2020). Effects of (+)-bicuculline, a GABAa receptor antagonist, on auditory steady state response in free-moving rats. PLoS ONE.

